# Making room for manoeuvre: addressing gender norms to strengthen the enabling environment for agricultural innovation

**DOI:** 10.1080/09614524.2020.1757624

**Published:** 2020-05-15

**Authors:** Lone Badstue, Marlène Elias, Victor Kommerell, Patti Petesch, Gordon Prain, Rhiannon Pyburn, Anya Umantseva

**Keywords:** Gender and diversity, Environment (built and natural) – Agriculture, Food security, Labour and livelihoods –Poverty reduction

## Abstract

Local gender norms constitute a critical component of the enabling (or disabling) environment for improved agricultural livelihoods – alongside policies, markets, and other institutional dimensions. Yet, they have been largely ignored in agricultural research for development. This viewpoint is based on many years of experience, including a recent major comparative research initiative, GENNOVATE, on how gender norms and agency interact to shape agricultural change at local levels. The evidence suggests that approaches which engage with normative dimensions of agricultural development and challenge underlying structures of inequality, are required to generate lasting gender-equitable development in agriculture and natural resource management.

## Introduction

Strong evidence and compelling arguments have been marshalled to demonstrate how addressing gender disparities in agriculture contributes to poverty reduction and food and nutrition security. Agricultural research and development interventions have sought to address “gender gaps” through sex-disaggregated data collection and analysis, and increased integration of gender considerations in project design, aiming to improve women’s access to new agricultural technologies, knowledge and inputs. Why then do ingrained patterns of gender inequality persist in so many agricultural contexts? What is constraining lasting change?

Progress has been achieved in agricultural research for development (AR4D) identifying and targeting women’s needs, thus seeking to address the visible symptoms of inequality. However, such approaches often overlook the ways in which social norms, attitudes, and distributions of power and resources differentially frame women’s and men’s perceptions of, and capacities to seize, opportunities. Inequalities remain, and are sometimes reinforced by AR4D, as those who are well positioned to take advantage of new opportunities do so, while others fall further behind. As Kantor (2013, 3) puts it:

These approaches can offer no assurance that women will be able to take advantage of or benefit from new opportunities or technologies because society’s understandings of what is acceptable for women and men to be, do, own and control may continue to impose barriers.

In this viewpoint we draw on recent research to argue for approaches that stimulate and build space for normative change as vital to processes of agricultural innovation that enhance gender equality. Insufficient appreciation of how underlying social institutions and structures, such as gender norms, perpetuate gendered inequalities means that interventions often fail to achieve lasting benefits for women. In the worst cases, they may inadvertently reinforce gender disparities, thus hampering progress towards Sustainable Development Goal (SDG) 5 – “empowering all women and girls and achieving gender equality” – and other SDGs. As we show, systematic concern for the strong normative influences on agrarian development will enhance the relevance and effectiveness of AR4D and its contributions to the SDGs.

## Gender norms as part of the context for agricultural and natural resource management interventions

Gender norms constitute the social rules that frame what is considered typical and appropriate for a woman and a man to be and do in their society. Across much of the world, gender norms attach submissive and reproductive roles to women, and authority and productive roles to men. These normative frameworks profoundly shape how women and men perceive and act on opportunities in their lives, as well as how institutions function at various scales. An often-used metaphor is that of an iceberg: like the base of an iceberg, gender norms are powerful, dynamic and mostly hidden, but they underpin what can be observed at the surface. More than other dimensions of social differentiation, such as ethnicity, caste or religion, expectations related to gender reach deeply into the private sphere and govern an individual’s most intimate relations (Ridgeway and Correll 2004).

Gender norms are learnt and internalised from a very young age and maintained and reproduced in different ways; for example, when we see others conform to and value these societal expectations, and perceive that our own social approval hinges on compliance (Bicchieri 2006). Social pressure, public surveillance and sanctioning practices also play important roles in maintaining norms.

Yet, social norms about gender are not static. They vary across contexts and over time. In their day-to-day lives women and men negotiate, resist and sometimes redefine confining dictates when they constrain or no longer hold much relevance. Other times, gender norms are invoked to demonstrate or encourage compliance or to maintain the status quo.

Representing deep beliefs and expectations of what is considered normal, dominating gender norms infiltrate everyday social life and practice, and are embedded in the institutions and structures that organise societies. Heise et al. (2019) show how gender norms shape different pathways to health outcomes, including through formal institutions and structures, and in the very health research system itself. Along similar lines, we hold that gender norms are part of the enabling (or disabling) environment for agricultural interventions and greatly influence *who* is able to learn about new things in agriculture, try them out, adopt or adapt them, and benefit from them – and who is not. Agricultural markets, extension services, agricultural development programmes and research systems are shaped by and tend to uphold dominating gender norms.

## GENNOVATE: gender norms, agency and innovation in agriculture and natural resource management

During 2014–18, a group of social scientists working within international AR4D carried out a global comparative research initiative – GENNOVATE – to analyse how local social contexts, and especially gender norms, condition who can (and cannot) access, adopt, and benefit from agricultural innovations (Badstue et al. 2018b).

**Figure 1.**
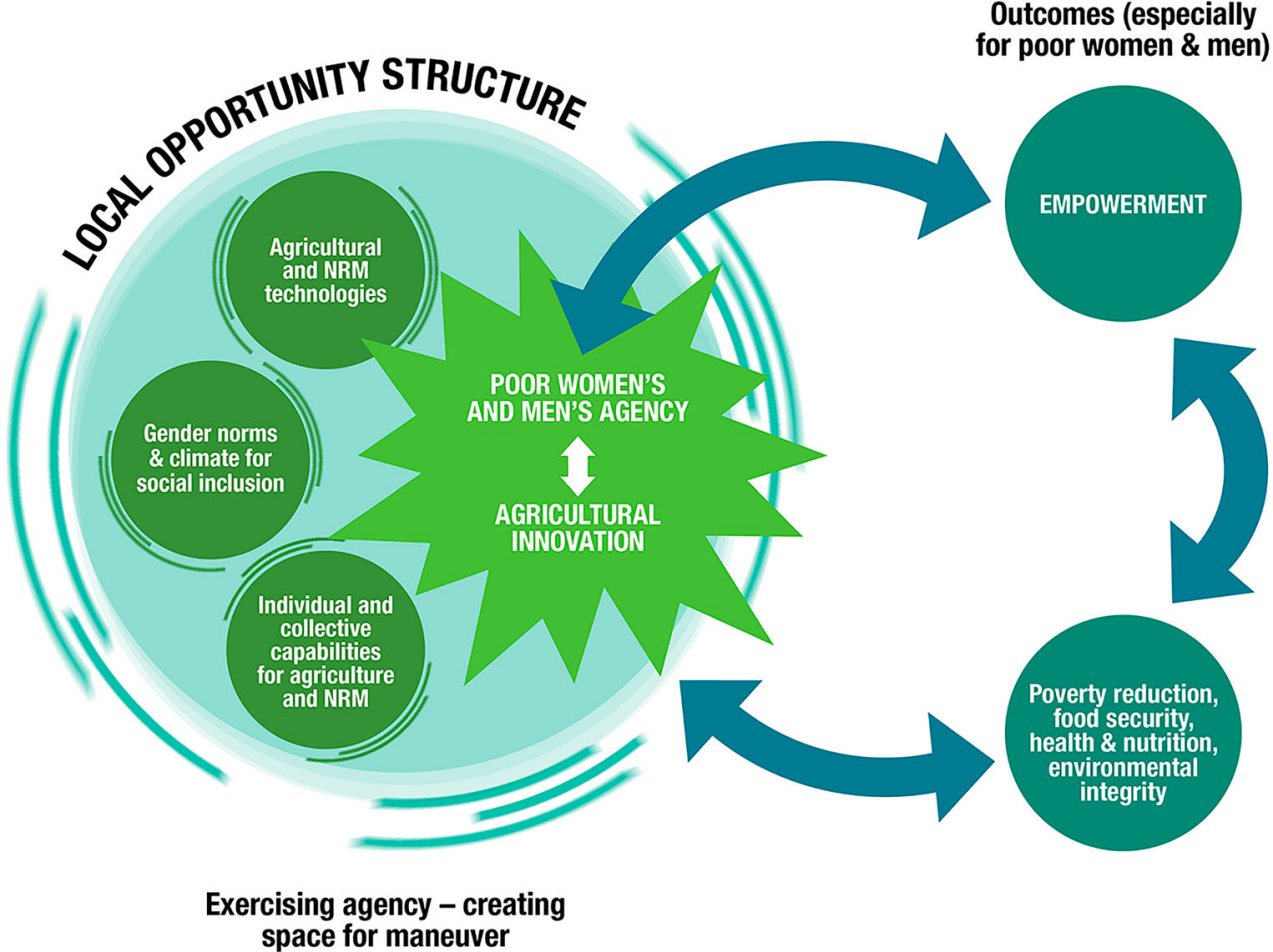
A framework for understanding the linkages between gender norms, agency and innovation in agriculture and NRM.

Innovation is understood as a social construct that can include technical, socio-economic, institutional or organisational change (Badstue et al. 2018a; Badstue et al. 2018b). Whether externally introduced or developed by farmers themselves, agricultural innovation not only requires strong agency (the ability to make strategic decisions concerning one’s own life and to act upon them), but is contextually embedded and shaped by gender norms as well as other dimensions of what can be described as the local opportunity structure. This comprises the specific combinations of agricultural and natural resource management (NRM) technologies, infrastructure, institutions, social organisation and other resources in a local context. Together, these dimensions set the conditions for whether and how local actors – women and men with different capacities to pursue their interests – search out space for manoeuvre to improve their lives (Figure 1, left side).

In the middle of the diagram, local women’s and men’s exercise of agency is illustrated with the shape of a spark – or an explosion, which pushes against the opportunity structure and existent normative practices, and eventually results in change in people’s ability to act and to drive institutional and structural change. Meanwhile, the right side of the figure calls attention to the links between expansion of agency and the process of empowerment and other desired outcomes, which, in turn, feed back into the local opportunity structure.

Many conceptions of innovation de-emphasise the importance of agency and how this may differ for women and men. Our model provides for diverse types of changes in the opportunity structure, illustrated with fuzzy lines, but emphasises the agentic “spark” that is indispensable for inclusive and empowering innovation processes. Factors such as new agricultural technologies, jobs, education or ICT may enlarge women’s agency without necessarily having much effect on the norms that underpin gender roles and relations.

### Illustrative research findings

GENNOVATE research teams conducted focus group discussions and individual interviews with more than 7,500 women and men from 137 communities in 26 countries across Africa, Asia, and Latin America. (Petesch et al. 2018a). Study participants reflected on questions such as:

– What qualities make a woman a good farmer? And a man a good farmer?
– What are the differences between a woman who is innovative and likes to try out new things and a man who is innovative?
– How would a typical couple in your village decide how much of the wife’s home garden produce to sell and how much to keep to feed the family? Would the wife decide? The husband? Would they decide together?

Here, we highlight findings that illustrate different interactions in the opportunity structure, with emphasis on the mutually influencing relationship between gender norms, and local women and men’s ability to exercise agency and innovate in their agricultural and NRM-based livelihoods.

Across study communities, men and women mainly report a growing capacity to take important decisions as well as declining poverty (Petesch et al. 2018c). While agricultural innovation is seen to contribute to these trends, the ability to innovate remains widely conceived as men’s sphere of action. Where acknowledged, women’s agricultural activities are widely framed as small endeavours or “helping men”. As part of the local opportunity structure, normative expectations often prevail that women should defer to men’s authority, shoulder the family’s housework and care burdens, and guard their physical mobility, social interactions, and use of resources.

Despite the prevalence of many restrictive norms, GENNOVATE also uncovered how women exercise agency to engage with innovations in agricultural production, post-harvest processing, and marketing across study geographies. Some women are innovating and influencing important agricultural decisions in their households, and actual practices in a village may be some distance from local norms that discourage women’s economic agency. However, processes whereby some norms relax while others remain restrictive, proved quite variable both within and across study communities.

In women’s focus groups from 43 diverse wheat growing communities, gender-related restrictions associated primarily with limited physical mobility and reproductive work burdens was the second-most frequently mentioned barrier to innovation by women, after lack of money/poverty (Badstue et al. 2017). In varied contexts, different norms also discourage women from doing certain agricultural tasks, such as land preparation or use of machinery, and they face barriers if they lack access to men’s labour or hired labour (Farnworth et al. 2019).

Gender norms also influence formal institutions in the local opportunity structure. For example, women and men alike testify that agricultural extension services continue to bypass most women. Women’s access to extension is often limited by household demands and constraints on their physical mobility and social interactions. An analysis of 336 innovative men and women’s experiences from 19 countries finds that although women appreciate extension services, only 26% consider these services significant for their innovation success, compared to 39% of male innovators (Badstue et al. 2018a). In cases from Nepal, women are increasingly managing farms due to high rates of male outmigration, but extension support often continued to be offered predominately to men (Farnworth et al. 2019).

GENNOVATE analyses especially forefront the fluidity of gender norms, and how they vary within and among communities. Norms relax and tighten as women and men move through their life cycle and change positions within their household; and they differ across caste, ethnic, religious and socio-economic groups (Cohen et al. 2016; Locke et al. 2017; Aregu et al. 2018; Petesch et al. 2018b). At the same time, young people spanning diverse contexts widely report strong gender inequalities in their opportunities to learn about and try out new farming practices (Elias et al. 2018).

Analyses from GENNOVATE bring to light how women’s innovation processes often require negotiation of local norms, and receive limited recognition and returns. Yet, selected cases also reveal contexts where local opportunity structures are benefitting from a catalytic mix of dynamic markets, infrastructure investments, men’s migration *and* more equitable gender norms for women’s productive roles, and these dynamics are driving local innovation and strong empowerment and poverty reduction (Petesch et al. 2018c). The diverse norms that hinder women’s economic participation may relax relatively quickly, and innovation in contexts of growing gender equality can unlock transformative processes of social change.

### Towards systemic change in agricultural research and development

If norms matter for agricultural development, how do we stimulate normative change and support the evolution of institutions that nurture more gender-equitable processes of agricultural innovation? Gender transformative approaches to research and development focus on fostering deep, structural and systemic change in gender-based power relations, at multiple levels, such as in households and communities, and various institutional domains (Hillenbrand et al. 2015; Galiè and Kantor 2016; Wong et al. 2019). Gender relations are the focus rather than men or women as independent entities. Pursuing gender transformative approaches requires pushing the agenda beyond merely reaching or benefiting women and men equally, to explicitly supporting initiatives that reduce institutional barriers to women’s empowerment and to gender equality, including through enhancing women’s access to and control over a range of resources, their voice in decision-making, and fostering a more equitable intra-household distribution of domestic and care work.

**Figure 2.**
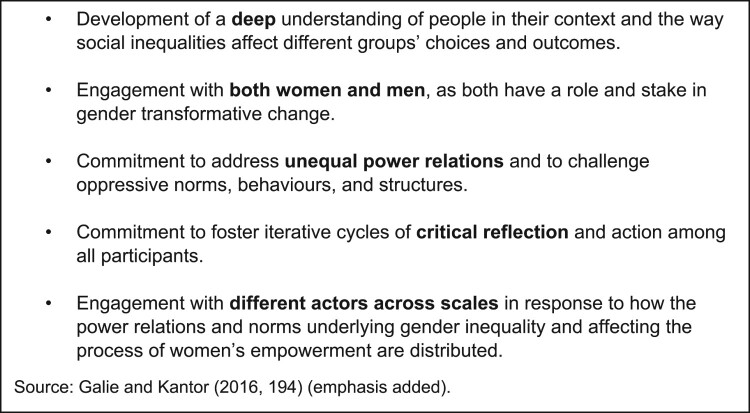
Core characteristics of gender transformative approaches (GTAs).

Gender transformative approaches are change-oriented: they identify, support learning from, and strengthen institutions and practices that support equality, and conversely, they challenge and change social structures and norms that justify and uphold the persistence of gender inequalities (Figure 2).

Reflexivity and institutional change are core pillars of gender transformative approaches. Reflexivity requires acknowledging that, as scientists operating within AR4D, we are part of the rural economy and the agricultural systems that (re)produce patriarchal norms and gender inequality. AR4D has long been complicit in overlooking women’s roles as (skilled) farmers and their contributions to natural resource management.

The need for more multi-faceted partnerships and intervention models to support both women and men to access opportunities is clear. For instance, an evidence review of gender interventions finds strong benefits from locally tailored projects that combined farmer groups, financial services, processing and storage technologies, and training; and while these programmes targeted women, they also “involved male partners and community leaders” (Buvinic, Furst-Nichols, and Courey Prior 2016, 40). Normative change requires coordinated shifts among community members in support of women’s economic independence, voice and leadership. The public health sector has developed valuable research and intervention designs that draw on social norms theory and community-based education and mobilisation strategies to reduce harmful practices, such as gender-based violence and female genital cutting (e.g. Cislaghi, Manji, and Heise 2018). In the field of AR4D, however, gender-transformative research is still relatively new territory, with great need for increased attention.

## Concluding remarks

Gender norms research is part of a wider shift in paradigms that examine and learn from the interdependent elements and evolution of local institutions, as well as the central role of local actors in processes of social change and development (Cunningham and Jenal 2016). This paradigm shift exemplifies the need for rigorous and inclusive learning initiatives to better understand and support local innovation processes that both poor women and men deem to be empowering.

To progress further, a transition from exploratory studies to applied research models on gender norms and institutional innovation is required. Components of an invigorated research agenda include: critical self-reflection and introspection among research institutions on the norms they bring to the research process; partnerships with civil society and other organisations with long-term, trusted local presence; engagement with both women and men from different social groups on the structures and mindsets that hinder and enable equality and local people’s empowerment; sufficient time and resources to accompany a process of social change; and mechanisms to scale advances made using gender transformative approaches. With these elements as part and parcel of agricultural research and development, agriculture and NRM could be a key axle for enhancing gender equality in rural livelihoods.
